# Microglia heterogeneity in health and disease

**DOI:** 10.1002/2211-5463.13735

**Published:** 2023-11-16

**Authors:** Shilauni Dadwal, Michael T. Heneka

**Affiliations:** ^1^ Luxembourg Centre for Systems Biomedicine University of Luxembourg Belval Luxembourg; ^2^ Division of Infectious Diseases and Immunology University of Massachusetts Medical School Worcester MA USA

**Keywords:** distribution, microglial origin, neurodegenerative diseases, regulation, subtypes

## Abstract

Microglia, the resident immune cells of the central nervous system (CNS), have received significant attention due to their critical roles in maintaining brain homeostasis and mediating cerebral immune responses. Understanding the origin of microglia has been a subject of great interest, and emerging evidence suggests that microglia consist of multiple subpopulations with unique molecular and functional characteristics. These subpopulations of microglia may exhibit specialized roles in response to different environmental cues as in disease conditions. The newfound understanding of microglial heterogeneity has significant implications for elucidating their roles in both physiological and pathological conditions. In the context of disease, microglia have been studied rigorously as they play a very important role in neuroinflammation. Dysregulated microglial activation and function contribute to chronic inflammation. Further exploration of microglial heterogeneity and their interactions with other cell types in the CNS will undoubtedly pave the way to novel therapeutic strategies targeting microglia‐mediated pathologies. In this review, we discuss the latest advances in the field of microglia research, focusing specifically on the origin and subpopulations of microglia, the populations of microglia types in the brains of patients with neurodegenerative diseases, and how microglia are regulated in the healthy CNS.

## Graphical abstract

Microglia, the CNS's resident immune cells, display significant heterogeneity, with distinct subpopulations responding to various environmental cues in both health and disease. This diversity has vital implications for understanding their roles, especially in neuroinflammation. Investigating microglial origin, subtypes in neurodegenerative diseases, and healthy CNS regulation offer promising therapeutic avenues for addressing microglia‐related pathologies.

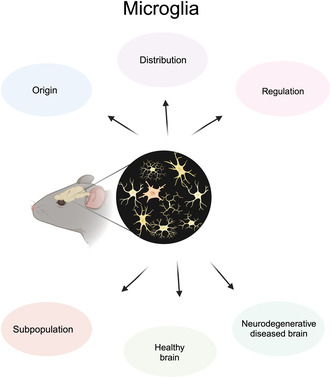

**Abbreviations**
AD: Alzheimer's disease
ALS: amyotrophic lateral sclerosis
APOE: apolipoprotein
APP: amyloid precursor protein
ASD: autism spectrum disorder
Aβ‐plaques: amyloid beta plaques
BDNF: brain‐derived neurotrophic factor
CCL4: C‐C Motif chemokine ligand 4
CCR9: CC Chemokine receptor type 9
CD40: cluster of Differentiation 4
CNS: central nervous system
CSF1: colony stimulating factor 1
CX3CR1: CX3C receptor 1
CyTOF: cytometry by time of flight
DAMs: disease‐associated microglia
EAE: experimental autoimmune encephalomyelitis
EGA: estimated gestational age
EGFP: enhanced green fluorescent protein
ETS: E‐twenty six
GABA: gamma‐aminobutyric acid
H3K27me3: H3 lysine 27 trimethylation
IBA1: ionized calcium‐binding adaptor molecule 1
IL10: interleukin 10
IL4: interleukin 4
Irf8.: interferon regulatory factor 8
KSPG: Keratan sulfate proteoglycan microglia
MGnD: neurodegenerative microglia
MS: multiple sclerosis
NGF: Nerve Growth Factor
P2RY12: purinergic receptor P2Y, G‐protein coupled 12
PD: Parkinson's disease
PGE2.: prostaglandin E2
PRC2: polycomb repressive complex 2
PRR: pattern recognition receptor
RMS: rostral migratory stream
SVZ: subventricular zone
TNF‐α: tumor necrosis factor‐alpha
TREM2: triggering receptor expressed on myeloid cells 2

The central nervous system (CNS), the most intricate human organ system, comprises billions of neurons coordinating the intricate interplay of thoughts, emotions, and physiological functions. However, the complex network requires constant surveillance and protection to maintain its delicate balance. So, in the brain, microglia serve as the guardians, playing a vital role in immune defense and maintaining cerebral homeostasis. As part of the innate immune system, microglia continuously monitor the CNS for potential internal and external threats [1, 2, 3]. In addition to their crucial role in defending the CNS against pathogens, microglia also contribute significantly to controlling neuronal proliferation, synapse formation, and elimination as well as debris clearance [4, 5, 6]. Moreover, they actively participate in remodeling neuronal circuits in postnatal mice [7]. Given the diverse functions, it is not surprising, given their diverse functions, that microglia represent a heterogeneous myeloid cell population, which is indispensable for the CNS in health and disease [8].

Considering the significance of microglia, it is important to explore their origin, their various subpopulations, and their precise mode of action. Microglia, as essential neuroglial cells, account for 5–20% of the entire glial population in mice while in humans, they constitute 0.5–16.6% of the total population [9].

In this article, we review the recent findings in the field of microglia research with a focus on their heterogeneity. The identification of distinct microglial subtypes and their unique functional characteristics have revolutionized our understanding of their roles in brain development, homeostasis, and disease processes. Understanding the modulation of these different microglial subpopulations will be key to develop precise therapeutic interventions targeting CNS innate immune mechanisms.

## Origin of microglia

Over the past 160 years, multiple hypotheses about the origin of microglia have emerged. W. Ford Robertson was the first to introduce the term “mesoglia”, a phagocytic element derived from the mesoderm, distinct from neurons and other CNS cells. In 1856, Virchow coined the term “neuroglia” (originally “Nervenkitt”), referring to these cells as “nerve‐glue,” which was later translated to “neuroglia” [10]. Santiago Ramon y Cajal, a prominent scientist in the field of neuroscience, renamed the cells the “third element of the nervous system.” A student of his, Pio del Río‐Hortega, continued working on these cells and made significant contributions to the understanding of the “third element of the nervous system.” Del Río‐Hortega studied the third element of the nervous system using silver carbonate impregnation staining. He redefined the concept of the “third element” based on its morphology and function, which he named “microglia cells.” Microglia cells are characterized as a small population of phagocytic and migratory immune cells in the CNS, distinguishing them from neurons, astrocytes, and oligodendroglia, which are of neuroectodermal origin [11].

In murine models, the precise origin of microglia has sparked controversies. It was believed that microglia are present during early development, suggesting that they originated from embryonic progenitors. Del Río‐Hortega proposed an additional possibility, that microglia could derive from meningeal macrophages. Another hypothesis during that era was that microglia could originate from blood monocytes [10, 11]. Ashwell and colleagues initially observed the presence of amoeboid microglia cells at E11.0 in the fetal mouse cerebellum and later in the rat forebrain [12, 13]. Subsequently, Sorokin and his colleagues detected macrophage precursors and macrophage‐like cells in the embryonic mesenchyme and blood vessels in rats starting from E10.5, highlighting the brain as the first organ to be colonized [14].

The origin of microglia was studied using genetic mapping, which revealed that microglia originate from yolk sac primitive macrophages [15, 16, 17, 18, 19]. During embryonic development in mice, between embryonic days E8.0 and E10.0, there is blood vessel formation and remodeling [20]. Around E7.0, precursor cells expressing vascular endothelial growth factor migrate from the primitive streak to the proximal yolk sac, where they form blood islands. These blood islands house the multilineage c‐kit^+^ erythromyeloid yolk sac precursor cells that give rise to microglia [21, 22]. The yolk sac precursor cells mature from A1 (CD45^+^ c‐kit^lo^ CX3CR1^−^ F4/80^−^) to A2 (CD45^+^ c‐kit^−^ CX3CR1^+^ F4/80^hi^) amoeboid macrophages in the blood islands and cephalic mesenchyme. Eventually, they acquire the phenotype of mature macrophages in the neuroepithelium by E10.5 [23]. Fate mapping studies using genetic targeting of hematopoietic precursors expressing the runt‐related transcription factor Runx1 between E6.5 and E10.5 have shown that yolk sac macrophages are specified between E7.0 and E7.5 [15]. An interesting study demonstrated that normal yolk sac hematopoiesis from E9.5 to 10.5 in Ncx‐1 knockout mice causes the absence of brain microglia progenitors. This supports the notion that brain recruitment of yolk sac progenitors depends on a functional circulatory system [15]. Microglia enter the developing brain through the leptomeninges and lateral ventricles at E9.5 and then distribute within the cortical walls from both directions. The speed of migration, proliferation rates, and maturation of microglia vary based on the region and developmental stage [15, 24, 25]. In early postnatal weeks of mouse development, there is an increase in the number of microglia cells. Subsequently, there is a gradual decrease in their number, reaching approximately 50% of the peak density. From Week 5 to 6, microglia density stabilizes [26].

In human fetuses, microglia‐like cells can be detected as early as 3 weeks of the estimated gestational age (EGA) [27]. By Week 4.5, amoeboid microglial cells migrate into the cerebral wall via the pial surface, ventricle, and choroid plexus [28, 29]. In the white matter, subplate, and cortical plate layers, radial and tangential migration was observed and then at 12–13 gestational weeks, the second wave of microglia was observed via the vasculature [29, 30]. Around Week 9, colonization of the spinal cord begins, and by Week 16, there is a significant influx and distribution of microglia throughout the entire CNS. It takes approximately 22 weeks for microglia to adopt a ramified form and to develop widely distributed processes. Importantly, well‐differentiated microglia are detected at 35 weeks of pregnancy [28, 30, 31, 32, 33]. These studies provide strong evidence that microglia originate from embryonic hematopoietic precursors that populate the CNS before birth and prior to bone marrow hematopoiesis. In zebrafish, yolk sac macrophages initially invade the entire cephalic mesenchyme and subsequently infiltrate epithelial tissues, including the brain. Additionally, other macrophages enter the blood circulation, indicating that colonization occurs independently of the blood circulation [34, 35]. Summarizing the current knowledge suggests the origin of microglia is from yolk sac primitive macrophages, and the colonization takes place before the formation of neuroectoderm‐derived cell types, such as astrocytes and oligodendrocytes. Microglia remain in the brain throughout life and self‐renew [15, 16, 17, 18, 19, 22, 25, 30, 36].

## Microglia subpopulations

In the CNS, microglia are non‐uniformly distributed, as observed by Lawson and colleagues using polyclonal antiserum targeting the F4/80 marker. Their study revealed that the telencephalon region has the highest density of microglia, followed by the diencephalon, mesencephalon, and rhombencephalon, which contain fewer microglia. Furthermore, the gray matter is more densely populated with microglia compared with the white matter [37]. These studies have proposed the existence of different microglial subtypes, each associated with a distinct molecular signature [38]. Understanding these potential subtypes will provide insights into the differential responses of microglia to intrinsic and extrinsic stimuli i.e.; microglia near Aβ‐plaques show a neurodegenerative profile controlled by TREM2‐APOE, and targeting APOE can revert them to a healthy state, reducing apoptotic neuron phagocytosis [39, 40, 41, 42, 43, 44].

What makes a cell subtype? Traditionally, a cell type is defined based on its host tissue, morphology, lineage, function, and molecular composition [45]. From a historical perspective, del Río‐Hortega already defined a microglial subtype known as “satellite microglia,” which are located in close proximity to neuronal cell bodies [46]. This early observation highlights the concept of microglial subtypes based on their distinct anatomical localization and association with specific cellular components or morphological structures. It is important to continue investigating and characterizing microglial subtypes to gain a deeper understanding of their functional diversity and associated molecular signatures. Microglia distribution in the CNS is not only variable concerning localization but also varies in morphology based on their association with different cellular components such as neuronal cell bodies, dendrites, axons, myelinated axons, and blood vessels. This variability is also reflected at the transcriptional level [47, 48]. Several studies have mounted evidence that microglia isolated from unchallenged adult murine brain exhibit variability in gene expression patterns depending on the brain regions which they were isolated from. Various markers such as CD40, CD11b, CD45, CD80, CD86, F4/80, Triggering Receptor Expressed on Myeloid Cells 2b (TREM2b), CX3CR1, and CCR9 show variable expression levels in microglia based on brain area and transcriptome analysis using preselected panels [49, 50]. Furthermore, recent research by Jordão and colleagues revealed the diversity of CNS‐associated macrophages in three subsets expressing high levels of Mrc1, Ms4at, Pf4, Stab1, Cbr2, CD163, and Fcrls. These subsets are associated with different CNS compartments, including the leptomeninges, choroid plexus, and perivascular space [51]. The microglia in the vicinity of these different types of neurons, as well as other glial cells such as astrocytes, oligodendrocytes, and progenitor cells, show distinct gene expression profiles under steady‐state conditions. Several subtypes of microglia have been identified based on their unique genomic, morphological, and function specialization.

These subtypes include satellite microglia, keratan sulfate proteoglycan‐microglia (KSPG)‐microglia, microglia supporting neurogenesis, Hox8b^+^ microglia, CD11c^+^ microglia, dark microglia, and TREM2‐positive‐microglia (Table 1). Satellite microglia interact with the axon initial segment in the healthy brain and lose the interaction upon injury. The markers required to identify satellite microglia are IBA1, CD11b, and CX3CR1; these microglia are frequently detected in the cortex and hippocampus [52, 53, 54]. KSPG‐microglia appear upon different insults, for example around motoneurons in Amyotrophic Lateral Sclerosis (ALS), and can be identified by the microglia marker IBA1, CR3, and CD11b. KSPG‐microglia are mainly present in the olfactory bulb, hippocampus, and brainstem [55, 56, 57]. Microglia‐supporting neurogenesis are essential for neuroblast survival and migration in the subventricular zone (SVZ)/rostral migratory stream (RMS), and their characteristics include the expression of IBA1^−^, isolectin B_4_^−^, CD68^−^, P2RY12^low^, pSTAT6^+^ cells as well as their ability to produce IL4 and IL10. Morphologically, these microglia are less ramified than microglia in neighboring brain cells and in the olfactory, subventricular zone, and rostral migratory stream, they can be identified by the expression of CX3CR1‐EGFP [58, 59, 60]. Hox8b^+^ microglia have a critical role in the functioning of the corticosteroid neuronal circuits. A deficiency of Hox8b^+^ microglia affects corticosteroid neuronal circuits negatively and leads to impaired grooming, anxiety, and altered social behaviors. Markers to identify Hox8b^+^ microglia are IBA1 and CD11b. These microglia are present in the olfactory bulb and cortex of the brain [61, 62, 63]. CD11c‐positive microglia promote myelination and neurogenesis in the neonatal brain and can be identified with markers IBA1, CD11c, CD45^low^, CX3CR1, and CCR2^null^. CD11c^+^ microglia cells are present in the corpus callosum and cerebellum [64]. Dark microglia, which interact with blood vessels and synapses, appear dark when detected by electron microscopy and can be identified by IBA1^low^, CX3CR1‐GFP^low^, CD11b, TREM2, and 4D4. Dark microglia can be found in the cortex, hippocampus, amygdala, and hypothalamus [65, 66]. These subtypes of microglia demonstrate diversity in their localization and functional roles. The role of TREM2 microglia in Alzheimer's disease (AD) is essential for neuroprotection. However, it is important to note that not all microglia express the TREM2 receptor. TREM2‐positive microglia are known for their survival and proliferation, and they tend to cluster around Aβ‐plaques in AD [67]. In murine models, TREM2 expression in microglia varies across different brain regions. The highest levels of TREM2 expression are found in the cingulate cortex and lateral entorhinal cortex, while much lower levels are observed in regions such as the hypothalamus and habenula. Interestingly, some regions, such as the circumventricular organs, completely lack TREM2 expression [68]. These regional differences in TREM2 expression are also observed in humans. Microarray data from 101 individuals revealed significant variations in TREM2 expression between different brain regions, particularly in the white matter and cerebellum. This suggests the presence of specific subtypes of microglia with varying roles in different brain regions, which may be relevant to the progression of AD and other neurological disorders [69]. Of note, the described pattern may vary and be dynamic over the entire lifetime and in particular during inflammatory challenges and activation.

**Table 1:** Microglia subpopulation in the CNS.

Microglial subpopulation	Markers	Specific brain region
Satellite microglia [46]	IBA1, CD11b, and CX3CR1	Cortex and hippocampus
Keratan sulfate proteoglycan‐microglia (KSPG)‐microglia [55, 56]	IBA1, CR3, and CD11b	Olfactory bulb, hippocampus, and brainstem
Microglia supporting neurogenesis [58]	IBA1^−^, isolectin B_4_^−^, CD68^−^, P2RY12^low^, pSTAT6^+^	Olfactory, subventricular zone, and rostral migratory stream
Hox8b^+^ microglia [61]	IBA1 and CD11b	Olfactory bulb and cortex of the brain
CD11c^+^ microglia [64]	IBA1, CD11c, CD45^low^, CX3CR1, and CCR2^null^	Corpus callosum and cerebellum
Dark microglia [65]	IBA1^low^, CX3CR1‐GFP^low^, CD11b, TREM2, and 4D4	The cortex, hippocampus, amygdala, and hypothalamus
TREM2‐positive‐microglia [67]	TREM2	Cingulate cortex and lateral entorhinal cortex

In addition to a subpopulation of microglia defined by differential gene expressions, there are differences in the microglia population between males and females [70, 71, 72, 73]. Microglial density in 13‐week‐old male mice is higher than in females, while the opposite holds true for 3‐week‐old mice, showcasing significant age‐ and gender‐related variation [74]. Hormones such as estradiol also play a major role in the gender‐dependent variation of microglia numbers [75, 76]. During early postnatal development, male mice have more microglia in the cortex, hippocampus, and amygdala compared with females. This is linked to increased expression of CC‐chemokine ligand (CCL) 20 and CCL4 due to testosterone, the primary masculinizing hormone, being aromatized to estradiol in the mouse brain. Adult female mice exhibit thicker microglia with longer processes in the hippocampus, amygdala, and cortex compared with male mice [76, 77].

## Microglia regulation

Microglia are crucial components of the neuroglial network in the healthy CNS. In the “homeostatic” state, microglia exhibit small cell bodies with ramified processes [78]. In this state, microglia do not overlap with the processes of neighboring cells, and each microglia cell actively surveys its immediate vicinity. While the soma of microglia remains stable, the processes constantly elongate and retract, allowing them to constantly explore the tissue environment widely. Upon stimulation by pathogen exposure, microglia rapidly retract their processes and become mobile effector cells [2]. The activation of microglia is triggered by various immune receptors, both endogenous and exogenous, that are present on their surface and collectively described as pattern recognition receptors (PRR) [79, 80, 81]. Examples of these PPRs include Toll‐like receptors (TLRs), scavenger receptors, CD36, and CD47, as well as numerous cytokine and chemokine receptors. Further surface receptors are involved in the regulation of microglial homeostasis, their interaction with neighboring cells, and immune reactivity (Table 2):

**Table 2:** Factors regulating microglia.

Microglial regulation	Function
CD200 [82]	Helps to maintain resting state
CX3CR1 [85]	Regulate microglia recruitment to the site of neuroinflammation
CD47 [84]	Neuronal protein sends “do not eat me” signals to microglia via CD172a/Sirp alpha interaction
PRC2 [91]	PRC2 enzyme catalyzes H3K27me3 modification
TREM2 [93, 94]	Role in phagocytosis of debris and reducing proinflammatory cytokines
CSF1 [95]	Regulate the survival of myeloid lineage
Runx1, ETS, PU.1, Irf8, Hoxb8 [15, 22]	Regulating differentiation processes during the embryonic development
C‐myb [97]	Essential for microglia health, regulates proliferation, and survival in the CNS
Ionotropic receptors [98, 99]	Calcium influx and the release of pro‐inflammatory molecules
Metabotropic receptors [98, 99]	Activate intracellular signaling cascades that contribute to microglial activation and inflammation
Nerve Growth Factor [100]	Regulate microglial activation and survival
Prostaglandins E2 [101]	Modulate microglial activation and pro‐inflammatory responses
Gamma‐aminobutyric acid (GABA) [105]	Modulate microglial activation and inflammatory responses
Glucocorticoids [106]	Suppresses microglia activation and inflammation via glucocorticoid receptor binding
Estrogen [106]	Modulates microglia activation, migration, and phagocytic activity
Brain‐derived Neurotropic factor (BDNF) [107]	Modulate microglial function by influencing their activation, proliferation, and release of pro‐inflammatory cytokines
Norepinephrine [108, 109]	Controls the release of inflammatory factors like interleukin 6 (IL‐6), interleukin 1β (IL‐1β), and tumor necrosis factor α (TNF‐α)
Histamine and serotonin [111]	Increases of calcium in the microglia

CD200 is a crucial molecule expressed on the surface of neurons, astrocytes, and oligodendrocytes. It serves as a receptor on microglia and macrophages, helping to maintain their resting state [82, 83, 84]. CX3CR1 is another essential molecule found on the surface of monocytes, macrophages, dendritic cells, and natural killer cells. Its ligand, fractalkine or neurotactin (CX3CL1), is present on neurons and interacts with microglia via CX3CR1. This interaction plays an important role in regulating microglia functions within the CNS [5, 85, 86, 87, 88, 89]. Loss of this interaction, as seen in animal models of PD and other neurodegenerative disorders, can lead to increased neuronal cell death [85]. CD47, expressed ubiquitously on neurons, transmits “do not eat me” signals to microglia through its interaction with CD172a/Sirp alpha [84, 90]. Ayata and colleagues showed that cerebellar microglia have a unique ability for clearance, while microglia in the striatum exhibit a homeostatic surveillance phenotype [90, 91]. It was also demonstrated that the suppression of clearance genes in striatal microglia is mediated by PRC2, an enzyme complex that catalyzes the repressive chromatin modification histone H3 lysine 27 trimethylation (H3K27me3). Removal of PRC2 in microglia results in enhanced clearance function in both the striatum and cerebral cortex even in the absence of dying neurons [91]. TREM2 is primarily known for its involvement in the phagocytosis of cellular debris and the downregulation of pro‐inflammatory cytokines [92]. Studies showed that the deletion of TREM2 showed identical phenotypes such as enhanced inflammatory cytokine production [93, 94]. Colony Stimulating Factor 1 (CSF1) plays a crucial role in regulating the survival of myeloid lineage cells in general [95]. Colony Stimulating Factor 1 binds to its receptor, CSF1R, and the absence of CSF1R results in a deficiency of several subsets of mononuclear phagocytes. Notably, in mice lacking CSF1R, microglia, are entirely absent [15, 96].

**Fig. 1:**
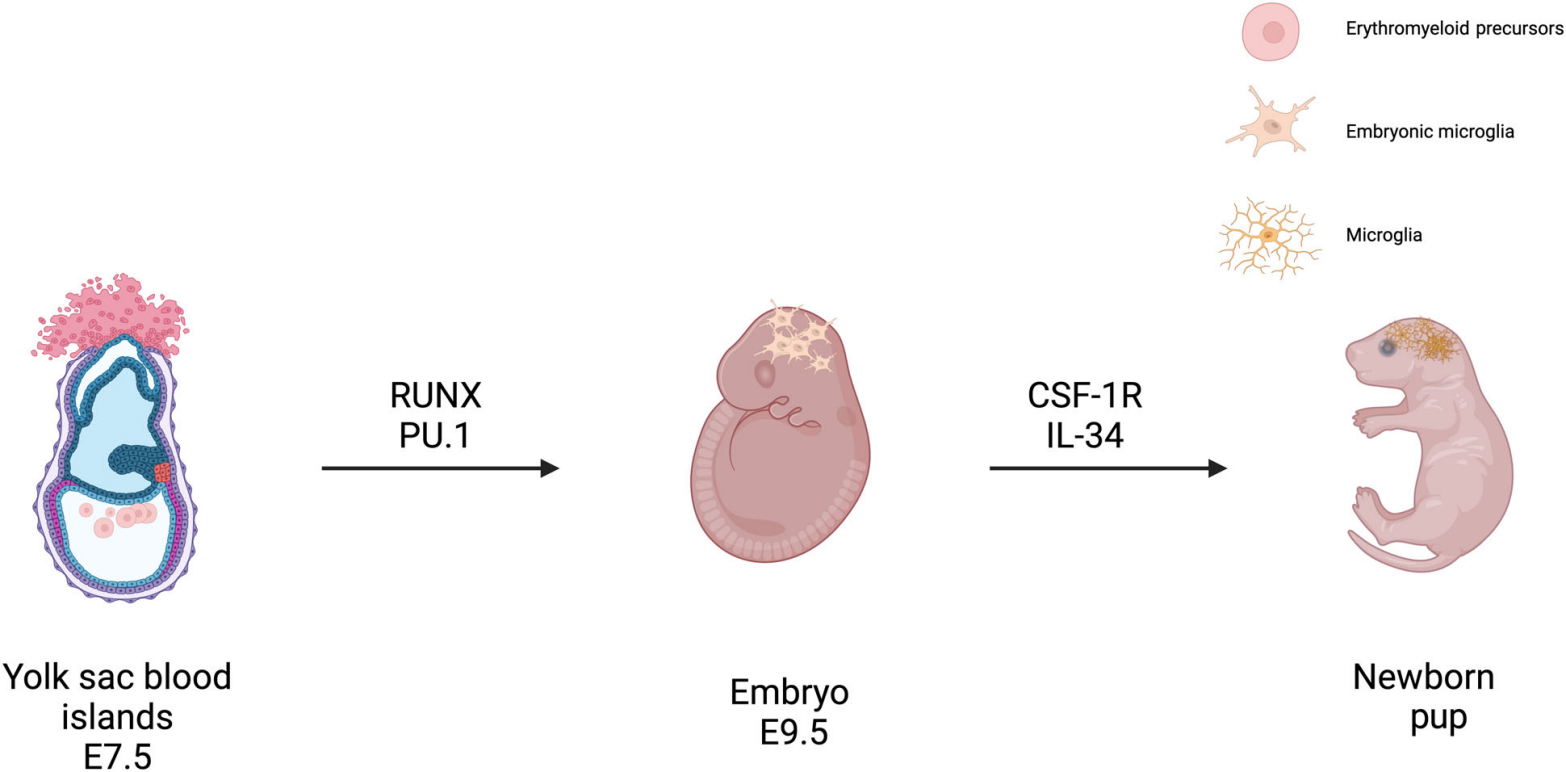
Origin of microglia in mice. Microglia stem from immature erythromyeloid precursors (EMPs) that depart the yolk sac blood island around E7.5, regulated by PU.1 and RUNX. By E9.5, these EMPs reach the neuroepithelium, giving rise to embryonic microglia, which mature into the fully developed form. Essential for mature microglia's growth are IL‐34 and CSF‐1, vital factors that support their proliferation.

Endogenous transcription factors, such as Runx1, ETS (E‐twenty six) family transcription factor PU.1 shown in Fig. 1, interferon regulatory factor 8 (Irf8), and Hoxb8, play crucial roles in regulating differentiation processes during the embryonic development [15, 22]. While c‐myb is not directly involved in microglia development, it plays a vital role in maintaining microglial homeostasis. C‐myb is an important regulator of cell proliferation and survival, and its function is essential for the normal functioning and maintenance of microglia in the healthy CNS [97]. ATP release in the CNS leads to various effects, including an inflammatory response, migration, and proliferation, ultimately resulting in a microglial activation [2]. This activation is mediated through the presence of purinergic receptors on the surface of microglia, including ionotropic receptors (P2X4, P2X7) and metabotropic receptors (P2Y1, P2Y2, and P2Y12) [98, 99]. Ionotropic purinergic receptors, such as P2X4 and P2X7, are involved in calcium influx and the release of pro‐inflammatory molecules. Metabotropic purinergic receptors, such as P2Y1, P2Y2, and P2Y12, activate intracellular signaling cascades contributing to microglial activation and inflammation. Nerve Growth Factor (NGF) can regulate microglial activation and survival. It has been shown to modulate microglial morphology and pro‐inflammatory responses [100]. Prostaglandins, including prostaglandin E2 (PGE2), can modulate microglial activation and pro‐inflammatory responses. They are synthesized by microglia through cyclooxygenase 1 and 2 and can act in an autocrine or paracrine manner [101].

Notably, microglia are well known to express receptors for neurotransmitters [102, 103, 104]. Gamma‐aminobutyric acid (GABA), an inhibitory neurotransmitter in the CNS, can also modulate microglial activation and inflammatory responses. Activation of GABA receptors on microglia has been shown to suppress their pro‐inflammatory phenotype, dampening the inflammatory response [105]. Glucocorticoids and estrogen have been shown to regulate microglia function. Glucocorticoids suppress microglia activation and pro‐inflammatory responses by binding to glucocorticoid receptors expressed on microglia. Estrogen modulates microglia activation, migration, and phagocytic activity [106]. Brain‐Derived Neurotrophic Factor (BDNF) can modulate microglial function by influencing their activation, proliferation, and release of pro‐inflammatory cytokines [107]. Norepinephrine regulates microglia and controls the release of inflammatory factors such as interleukin 6 (IL‐6), interleukin 1β (IL‐1β), and tumor necrosis factor α (TNF‐α). This regulation impacts neuroinflammation, neuropathic pain, anxiety, and depression [108, 109, 110]. Histamine and Serotonin induce the increase of calcium in the microglia [111]. Microglia and neurons communicate through receptors for various neurotransmitters, enabling neurons in specific brain regions to influence microglia heterogeneity based on their comcombined neurotransmitter profile. The communication between microglia and neurons via neurotransmitter receptors may be crucial for brain region‐specific differences, given variances in neuron‐derived transmitter profiles.

## Microglia in disease

Emerging research has shown that microglia represent a highly heterogeneous population composed of distinct subpopulations with diverse functions (Table 1). Dysregulation of these subpopulations has been implicated in various neurological and neurodegenerative diseases. Here are some examples of microglial subpopulations involved in specific diseases: Activation of microglia in brain diseases mostly functions through the ligation of PRRs, including the Toll‐like receptor family, the scavenger receptors, CD47, CD36, and several others that cooperate to induce downstream immune signaling pathways [79, 80, 81]. Various cytokines, such as interleukin‐1 beta (IL‐1β) and tumor necrosis factor‐alpha (TNF‐α), can modulate microglial activation and immune responses. They can be released by microglia themselves or by other cells in the CNS [112]. Consequently, microglia adopt disease‐specific states (Table 3), which still must be defined in greater detail, as most of the existing literature is of cross‐sectional nature and may not fully account for highly dynamic changes along the respective disease trajectories. Nevertheless, AD is linked to amyloid‐beta plaques from increased amyloid precursor protein (APP) production or clearance issues and hyperphosphorylated tau protein buildup [113, 114, 115]. Disease‐associated microglia (DAMs) and Neurodegenerative microglia (MGnD) represent a subset of microglia with heightened expression of AD‐risk‐associated genes (ApoE, Trem2, and Clec7a) and functional activation of the TREM2‐APOE pathway [40, 42]. Disease‐associated microglia is associated with protective phagocytosis, and MGnD is a dysfunctional microglial phenotype [116]. While many genes differ in DAM and MGnD between mice and humans, they share some similarities. Notably, both human microglia and activated mouse microglia show increased APOE expression, along with a reduced TREM2 expression [42, 117, 118]. They play a role in amyloid‐beta plaque clearance and may have both protective and harmful effects on AD pathogenesis [40]. In AD, the CD33 transmembrane receptor is mainly expressed by microglia, which regulates innate immune responses [119, 120]. Parkinson's disease (PD, a movement disorder caused by the degeneration of dopaminergic neurons) is characterized by motor impairment and the presence of intraneuronal inclusions called Lewy bodies, which represent aggregates of misfolded alpha‐synuclein [121]. During the course of PD, reactive microglia also become activated by alpha‐synuclein and release pro‐inflammatory cytokines and oxidative stress‐inducing factors, contributing to PD‐associated neuroinflammation and likely causing dopaminergic neuron loss [122, 123]. Multiple Sclerosis (MS) is characterized by multifocal white matter lesions. Experimental Autoimmune Encephalomyelitis (EAE) serves as an animal model for studying inflammatory demyelination disease [124, 125, 126]. Both neuroinflammatory and neurodegenerative models develop double‐positive TNF‐alpha‐ and GM‐CSF‐producing cells, this subset abundance correlated best with the height of neuroinflammatory condition in the MS model [124]. Within EAE lesion sites, three distinct subtypes, namely daMG2, daMG3, and daMG4, have been identified. These subtypes exhibit variations in the expression of particular chemokines and cytokines, and they also exert differing effects on homeostatic markers such as P2RY12 and TMEM119 [51]. The expression of 5D4‐KSPG is elevated within a specific subgroup of microglia that are positive for IBA1/CD11b in the context of amyotrophic lateral sclerosis (ALS) and in a Wallerian degeneration mouse model for spinal cord injury [57, 127, 128]. In stroke, pro‐inflammatory microglia can become activated and exhibit a pro‐inflammatory phenotype. These microglia produce inflammatory cytokines and reactive oxygen species, contributing to secondary brain damage [129]. In autism spectrum disorder (ASD), microglia show impaired synaptic pruning. These microglia fail to efficiently eliminate excessive synapses during brain development, potentially leading to disrupted or dysfunctional neural circuits and subsequently altered connectivity [130]. In brain tumors, the analysis of activated microglia using CyTOF, which is a single‐cell‐based immune phenotyping technique that relies on time‐of‐flight mass cytometry, has revealed significant differential expressions of HLA‐DR, TREM2, and APOE. [131].

**Table 3:** Microglia in disease.

Microglia identity	Present in
Disease‐associated microglia (DAM) & Neurodegenerative microglia (MGnD) [40, 42]	Alzheimer's disease AD model
Reactive microglia [122]	Parkinson's disease Alzheimer's disease PD model
Double‐positive TNF‐alpha‐ and GM‐CSF‐producing cells [124]	Multiple sclerosis MS model
daMG2, daMG3, and daMG4 [51]	Expexrimental autoimmune encephalomyelitis EAE model
5D4‐KSPG [57]	Amyotrophic lateral sclerosis ALS model

In summary, microglia are diverse and dynamic cells with important functions in the CNS and during CNS disorders. Understanding their origin, distribution, and subpopulations is crucial for unraveling their roles in both healthy and diseased conditions. Further research in this field will contribute to a deeper understanding of microglial biology and help to identify potential therapeutic interventions for CNS disorders.

## Conflict of interest

The authors declare no conflict of interest.

## Author contributions

SD authored the manuscript, and MTH edited and enhanced it, contributing to its refinement.
